# Genome-wide association study of *Klebsiella pneumoniae* identifies variations linked to carbapenems resistance

**DOI:** 10.3389/fmicb.2022.997769

**Published:** 2022-11-01

**Authors:** Na Pei, Wanying Sun, Jingxuan He, Yanming Li, Xia Chen, Tianzhu Liang, Karsten Kristiansen, Wenen Liu, Junhua Li

**Affiliations:** ^1^BGI-Shenzhen, Shenzhen, China; ^2^Laboratory of Genomics and Molecular Biomedicine, Department of Biology, University of Copenhagen, Copenhagen, Denmark; ^3^Department of Clinical Laboratory, Xiangya Hospital, Central South University, Changsha, China; ^4^Shenzhen Key Laboratory of Unknown Pathogen Identification, Shenzhen, China

**Keywords:** *Klebsiella pneumoniae*, carbapenems, genome-wide association study, drug resistance, variations

## Abstract

*Klebsiella pneumoniae* (KP) is one of the microorganisms that can acquire carbapenem-resistance (CR), and few antimicrobial therapy options exist for infections caused by Carbapenem-Resistant KP (CRKP). In recent years, with the increase of carbapenem resistance rates, treating CRKP has become a serious public health threat in clinical practice. We have collected 2,035 clinical KP isolates from a tertiary hospital in China. Whole genome sequencing data coupled with their binary antimicrobial susceptibility testing data were obtained to conduct the genome-wide association study using a bayesian-based method, including single nucleotide polymorphisms (SNPs) and genes. We identified 28 and 37 potential maker genes associated with imipenem and meropenem resistance, respectively. Among which 19 of them were selected in both drugs by genome-wide association study (GWAS), 11 genes among them were simultaneously validated in independent datasets. These genes were likely related to biofilm formation, efflux pump, and DNA repairing. Moreover, we identified 13 significant CR related SNPs in imipenem or meropenem, with one SNP located in the non-coding region and validated in the independent datasets. Our study indicates complex mechanisms of carbapenems resistance and further investigation of CRKP-related factors are warranted to better understand their contributions to carbapenems resistance. These identified biomarkers may provide targets for future drug interventions or treatments.

## Introduction

*Klebsiella pneumoniae* (belonging to *Enterobacteriaceae*) is one of the most common opportunistic pathogens and responsible for approximately one-third of Gram-negative bacterial infections (Navon-Venezia et al., 2017). It mainly causes urinary tract infections, pneumonia, bacteremia, and other nosocomial bacterial infections in people with weakened immune functions rather than healthy people (Podschun and Ullmann, 1998). In the past few decades, KP has evolved a type of hypervirulent strains, which can even cause liver abscess invasive infection syndrome in healthy people with normal immune function, and lead to complicated bacteremia, meningitis, endophthalmitis or necrotizing fasciitis simultaneously (Siu et al., 2012). Besides, the antibiotic resistance of KP also makes treatment more difficult.

Carbapenem-Resistant KP is classified as critical-priority bacteria in the WHO priority list of antibiotic-resistant bacteria. It indicates that it is pressing to propose a new KP antibiotic resistance solution. Carbapenem antibiotics mainly include imipenem (IPM), meropenem (MEM), and ertapenem, which have a wide antibacterial spectrum and strong bactericidal activity, and are currently the most effective drugs in clinical practice. Studies on their drug resistance mechanism mainly include the following aspects. The most important mechanism is that CRKP can produce β-lactamases that can hydrolyze most β-lactams antibiotics including carbapenems. Carbapenemases carried by KP are commonly found in genes *bla*_KPC_, *bla*_IMP_, *bla*_VIM_, and *bla*_NDM_ (Wyres and Holt, 2016). Furthermore, alterations in outer membrane permeability mediated by the loss of porins can cause reduced susceptibility to carbapenems (Martinez-Martinez, 2008). The overexpression of efflux pumps is also one of the mechanisms in the imipenem and meropenem resistance (Codjoe and Donkor, 2017). Meanwhile, in addition to these common mechanisms, other less reported carbapenem resistance studies have also been reported. It has been reported that the alteration in lipopolysaccharide (LPS) levels can also cause resistance to imipenem in Enterobacter aerogenes (Vieira et al., 2016). The mechanism of meropenem resistance has been widely reported by the production of carbapenemases and sometimes together with the overexpression of efflux pumps. Outer membrane permeability involving ompK35/36 has also been reported to be responsible for the unexplained resistance. However, in recent years, in addition to the cause of missing resistant mobile components and detection methods, more and more phenotypically carbapenem resistant KP have no corresponding resistance determinants explained in clinical practice. As a result, whether there are potential new genes or variations determinants is still unknown and searching for new resistant markers related to carbapenems resistance becomes necessary.

In recent years, with the development of sequencing technology, researchers can obtain more affordable and high-throughput bacterial genome sequencing results, making the idea of bacterial genome-wide association study (BGWAS) easier to be implemented. Some researchers have successfully used BGWAS to identify important adaptive traits associated with phenotypes. The pioneers conducted BGWAS in Campylobacter and identified specific factors related to vitamin B5 synthesis (Sheppard et al., 2013). Since then, dozens of studies have been published, and nearly 70 percent of them have focused on anti-resistance study. Francesc Coll et al. conducted a study on 6,465 multi-and extensively drug-resistant *Mycobacterium tuberculosis* clinical isolates and found new candidate genes and epistatic relationships (Coll et al., 2018). Maha R. Farhat et al. assessed the genome-wide association between mutations in the genes or non-coding regions and drug resistance in a 1,452 clinical *Mycobacterium tuberculosis* isolates and found 13 association non-standard loci (Farhat et al., 2019). Similar studies have been done in *Escherichia coli* and identified phylogroup-specific genes that have diverse functions including antimicrobial resistance (Mageiros et al., 2021). Thanks to the BGWAS, we can get insights in the field of antibiotic resistance and pathogenesis (Power et al., 2017). These insights will benefit identifying molecular targets for drug and vaccine development. However, due to the lack of genomic data combined phenotypic data with large sample size of KP, clone divergence of KP population, frequent homologous recombination, and complex resistance mechanisms, GWAS studies related to antibiotic resistance of KP have been rarely reported. Existing KP studies have focused on the use of genome-wide data by association analysis and machine learning to predict drug resistance phenotypes (Macesic et al., 2020). Exploring the discovery of KP drug resistance determinants studies by GWAS is imperative.

In this study, we describe the population structure of the 2,035 KP clinical isolates and try to identify genomics features statistically associated with carbapenems resistance in the KP using a bayesian-based GWAS method, including SNPs and genes. The large sample size increases the statistical power of BGWAS in our study. We also validated our findings in an independent public data set. We report 11 genes and one non-coding region SNP associated with resistance and investigate their role in carbapenems resistance. Our study highlights the potential genomics variations that may contribute carbapenems resistance.

## Materials and methods

### Phenotype and genotype data

2,035 clinical KP isolates were collected from a tertiary hospital, central of China between 2013 and 2018. The study was approved by the ethics committees under tracking numbers of 201806861 and BGI-IRB 18061. Antimicrobial susceptibility of imipenem and meropenem were tested by the Vitek 2 system (bio Mérieux, Marcy l’Étoile, France) and Kirby-Bauer disk diffusion test following the Clinical and Laboratory Standards Institute (CLSI) 2021 guidelines. *Escherichia coli* ATCC25922 and *Pseudomonas aeruginosa* ATCC27853 were used as controls. Strain names and a full list of MICs are given in Supplementary Table S1. Genomic DNA was extracted using the TIANamp Bacteria DNA kit (Tiangen-Biotech, Beijing, China) according to the manufacturer’s instructions. Samples were sequenced on BGISEQ-500 platform using 150-nt paired-end runs. The sequences data can be found at https://db.cngb.org/search/project/CNP0001198.

### Quality control, variant calling, and population structure

Fastp v0.14.0 (Chen et al., 2018; −5–3-q 20-l 30-c) and SOAPnuke v1.5.6 (Bankevich et al., 2012; default parameters) were employed for discarding the adapter, low quality end (quality ≤ 20). 2.5 million reads per sample were selected randomly by an in-house script and used for assembling genomes by Shovill v0.9.0. Samples whose genome size was outside of 5.0–6.5 Mbp, and GC content was outside of 40–60% were discarded. Also, we discarded isolates with the identity of KP less than 99.9% using mOTU (v 1.1.1) or Metaphylan (v 2.6.0). We aligned the clean reads to the reference KP isolate HS11286 (GCF_000240185.1) and call variants by Snippy v3.2-dev^1^ using default parameters. Core SNP alignments were used to remove recombination sites by Gubbins v2.3.1. The phylogenetic tree was generated by IQ-TREE v1.6.8 (Nguyen et al., 2015).

### Annotation and pan-genome gene matrix

Multi-locus sequence typing (MLST) was determined using the “*Klebsiella pneumoniae*” database from PubMLST (Jolley et al., 2018). Kaptive version 0.7.3 was used to identify Klebsiella capsule synthesis loci (K-Locus) from whole genome data (Wyres et al., 2016). Antibiotic resistance determinants were identified by ResFinder version 4.0 (Camacho et al., 2009; Bortolaia et al., 2020). Porin genes, ompK36 and ompK37, were also detected using PointFinder (Zankari et al., 2017). Prokka (Seemann, 2014)(--minlen 100) was performed to annotate the assembled contigs, and the pan-genome profile was generated by Roary v3.11.2 (Page et al., 2015). We generated a gene presence/absence matrix and combined it with the phenotypic data into the treeWAS-formatted files for GWAS.

### Genome-wide association analysis of SNPs and genes

TreeWAS (Collins and Didelot, 2018), a Bayesian-based GWAS approach, was used to identify novel mutations and genes with a permutation *p* value threshold of <0.01 to assess significance. Three complementary association scores of treeWAS were performed to enhance statistical capabilities. A genome-wide association was considered statistically significant if the *p* value for any SNP or gene was less than the Bonferroni-corrected significance threshold. All the genes and SNPs selected from treeWAS were considered as antibiotic resistant related candidates.

### Validate data set

We performed the validation study using data from two previous studies (Long et al., 2017; Nguyen et al., 2018). 1,000 KP isolates with the curated binary phenotype including 315 resistance and 685 susceptible isolates were selected from the study for IPM, and 325 resistance and 673 susceptible isolates for MEM, respectively (Supplementary Table S2). Sequence Read Archive (SRA) was downloaded from the public database. The clone groups and resistance profiles were not selected in the validated data set. The raw reads then undergo quality control, assembly with the same method as the data described above. All genes selected in test data with treeWAS software were performed fisher test in both validate data set and the combination of the test and validate data set. A locus was considered validated if the value of *p* < 0.01 and OR > 1.

## Results

### Phenotype and genotype characteristics of KP isolates

Clinical KP isolates were collected from a tertiary hospital, central of China with matched phenotype information and sequenced in our previous study (Pei et al., 2022). W carried out a GWAS analysis to determine genetic differences between drug resistant and susceptible isolates (Supplementary Table S1). Of the 2,035 isolates, 2,033 belongs to the IPM data set and 1,017 to the MEM. One strain only had MEM and the other had neither phenotypic data. 426 isolates were IPM resistant, 10 isolates were intermediate and 1,597 were IPM susceptible. 317 isolates were MEM resistant, 5 isolates were intermediate and 695 were MEM susceptible. These isolates were from various sample sources, including respiratory secretion, blood, drainage and puncture fluid, urine, wound secretion, digestive system secretion, abscess, and other sites. Most of these samples were isolated from respiratory secretion with the ratio being 55.6 and 56% in IPM and MEM datasets, respectively. 347 sequence types (STs) were identified by MLST typing in IPM dataset. The most prevalent ST was ST11 (*n* = 393), followed by ST23 (*n* = 203), ST37 (*n* = 76), and ST15 (*n* = 62). There were also 56 ST86 and 40 ST65 hypervirulent KP isolates in our data. Isolates in MEM dataset are divided into 223 STs, in which ST11 contains most of these strains (*n* = 284) either, followed by ST23 (*n* = 79) and ST37 (*n* = 34). ST11 also contains the highest proportion of resistant strains in which 92.1% (*n* = 362) and 93.9% (*n* = 265) are for IPM and MEM. A majority of STs are with a frequency of less than 3%, with 1,299 and 620 for IPM and MEM, respectively. The K-Locus sequence types were also investigated. 89 and 81 K-Locus were identified in IPM and MEM datasets, respectively. KL47 was the most common K-Locus followed by KL1in both data set (Table 1). Furthermore, we found IncF dominate the replicon type of the plasmid (*n* = 1909 and *n* = 958 in IPM and MEM) followed by replicon type Col. The detailed information can be found in Supplementary Table S1. All the isolates belong to the KP species complex (KpSC) KPI. Detailed clinical, phenotype and genotype information of the isolates were listed in Supplementary Table S1.

**Table 1:** Diversity and MLST information of the two carbapenems.

	IPM^a^ (*n* = 2,033)	MEM^b^ (*n* = 1,017)
No. of STs	347	223
No. of isolates (%) per ST^c^		
ST11	393 (19.3)	284 (27.9)
ST23	203 (10.0)	79 (7.8)
ST37	76 (3.7)	34 (3.3)
ST15	62 (3.0)	-
Others(<3%)	1,299	620
No. of isolates (%) per K-Locus		
KL47^d^	239 (11.8)	153 (68.6)
KL1	229 (11.3)	93 (9.1)
KL2	182 (9.0)	86 (8.5)
KL64	149 (7.3)	130 (12.8)
KL102	64 (3.1)	-
Others(<3%)	1,170	555
No. of isolates (%) per sample type		
Respiratory secretion	1,131 (55.6)	570 (56)
Blood	272 (13.4)	125 (12.3)
Drainage & Puncture fluid	200 (9.8)	90 (8.8)
Urine	161 (7.9)	92 (9.0)
Others(<90)	269	140

a IPM, imipenem.

b MEM, meropenem.

c ST, sequence type.

d KL, K-Locus.

### Genomic diversity and resistant phenotype explanation for the known carbapenems resistance genes

By whole-genome sequencing and *de novo* assembly, we identified 332,343 unique genetic core SNPs in the 2,033 IPM KP genomes, in which 296,450 SNPs (89.2%) are in coding regions. 119,676 (36%) are in only one of the 2,033 genomes and more than half of SNPs (187,926,63.4%) in coding regions were synonymous. As for the MEM dataset, 167,498 SNPs are synonymous variants which account for 65.7% of the 254,803 coding region SNPs, while 30,464 SNPs were in the non-coding region. We also identified 56,593 pan genes from 2,033 genomes in IPM dataset. We found 3,618 core genes exist in over 95% of strains. For 1,017 MEM dataset, we found 42,692 pan genes. 36,546 genes exist in no more than 15% of strains. Furthermore, 3,756 genes are core genes, which exist in over 95% of strains (Supplementary Figure S1). Both results showed that most of the genes were present in a small number of samples, revealing that the KP genomes were highly diverse.

**Figure 1:**
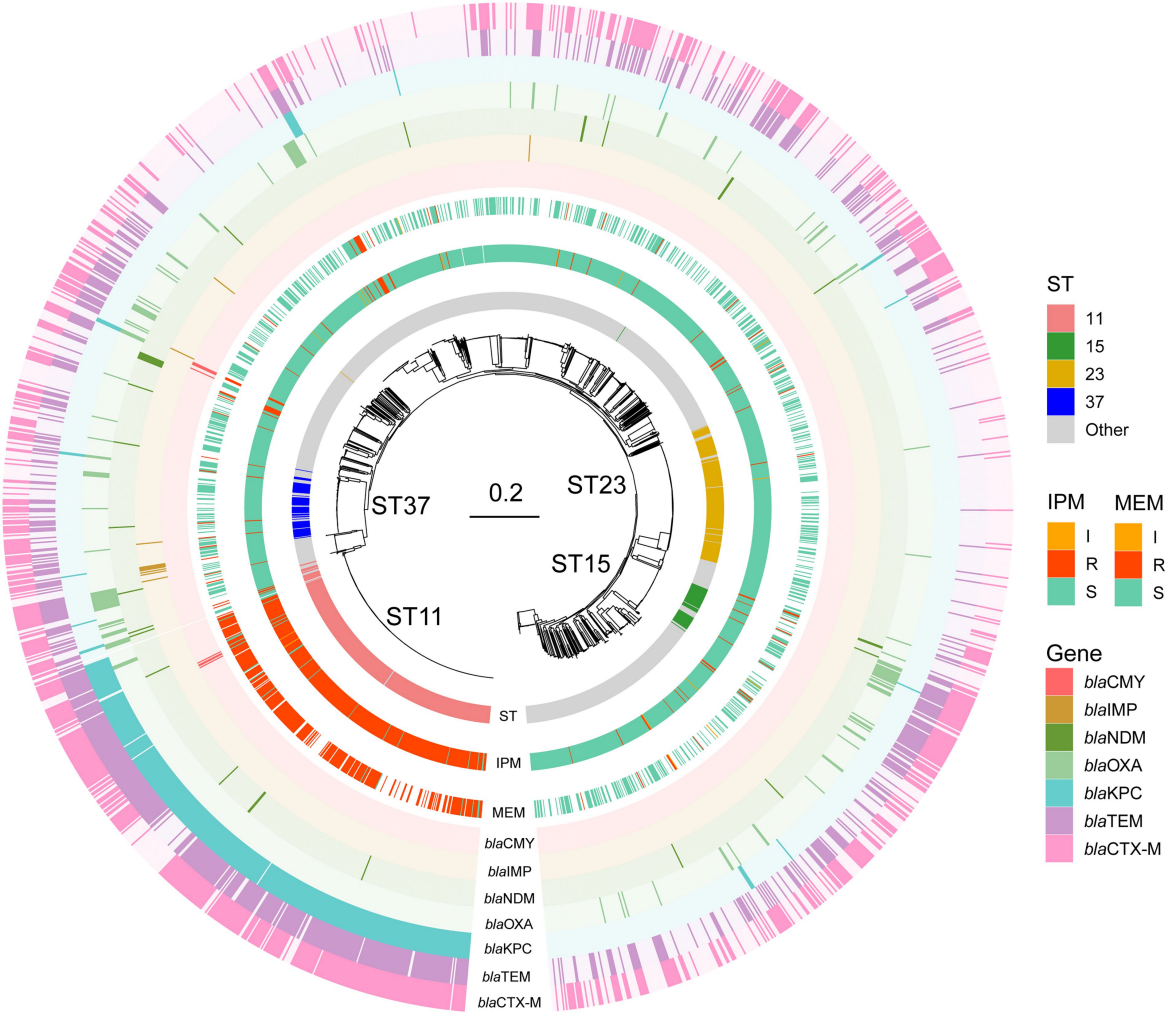
Phylogenetic tree, sequence type, and phenotype of the KP isolates for the two carbapenems. STs are shown in the inner ring and the most common STs ST11, ST23, ST37, and ST15 were marked. IPM and MEM phenotypes are in the middle ring. IPM: imipenem, MEM: meropenem. I, R, and S represent intermediate, resistant, and susceptible, respectively. In the outer ring, seven bla genes are labeled by different colors. Bla genes: beta-lactam resistance genes.

We examined the proportions of known beta-lactam resistance genes which may contribute to the carbapenem resistance in the resistance phenotype. Seven beta-lactam resistance determinants that could be relevant to carbapenem resistance reported by previous studies were searched and selected in the genomes including *bla*_NDM_, *bla*_KPC_, *bla*_IMP_, *bla*_OXA_ (Poirel et al., 2004), *bla*_CTX-M_ (Poirel et al., 2019), *bla*_CMY_ (Kim et al., 2006), and *bla*_TEM_ (Ripabelli et al., 2020). All the Odds Ratio (OR) of these *bla* genes >1 except *bla*_OXA_ and *bla*_IMP_ in IPM indicated an increased occurrence of carbapenems resistance. *Bla*_KPC_ (only *Bla*_KPC-2_ in our data) got the highest specificity of 0.87 and 0.88 in IPM and MEM, respectively. *Bla*_NDM_ including NDM1 and NDM5 has the highest sensitivity in both IPM and MEM. *Bla*_CTX-M_ got the lowest sensitivity of 0.62 and 0.63 in IPM and MEM. *Bla*_IMP_ and *Bla*_CMY_ both showed a low specificity in IPM and MEM (Table 2). No mutations or known mutations were found in ompK35, and point mutations of ompK36 and ompK37 were also summarized in Supplementary Table S1. Seven genomes have no carbapenems resistance genes detected in IPM and MEM data with a carbapenems resistant phenotype, indicating a more complex mechanism may exist. *Bla*_KPC_ has the majority of ST 11 genomes (*n* = 371,93.5%). *Bla*_CTX-M_ have the largest sample size (*n* = 497) followed by *bla*_TEM_ and *bla*_KPC_ and evenly distributed throughout the data (Figure 1).

**Table 2:** Resistance phenotype explained for the known carbapenems resistance genes.

Drugs	Gene^b^	Odds Ratio	*p* Value	Specificity	Sensitivity
IPM^a^	*bla*_NDM-1_	40.36 [9.42, 172.81]	<0.0001	0.05 [0.03, 0.07]	1.00 [1,1]
	*bla*_NDM-5_	55.79 [3.18, 978.84]	0.0059	0.02 [0, 0.03]	1.00 [1, 1]
	*bla*_KPC-2_	541.27 [318.56, 919.69]	<0.0001	0.87 [0.84, 0.9]	0.99 [0.98, 0.99]
	*bla*_IMP_	0.81 [0.18, 3.78]	0.7918	0.00 [0, 0.01]	0.99 [0.99, 1]
	*bla*_OXA_	0.69 [0.43, 1.12]	0.1306	0.05 [0.03, 0.07]	0.93 [0.92, 0.94]
	*bla*_CTX-M_	5.12 [4.02, 6.53]	<0.0001	0.76 [0.72, 0.8]	0.62 [0.59, 0.64]
	*bla*_CMY_	11.13 [2.24, 55.33]	0.0032	0.01 [0, 0.02]	1.00 [1, 1]
	*bla*_TEM_	9.58 [7.39, 12.43]	<0.0001	0.81 [0.77, 0.84]	0.70 [0.68, 0.72]
MEM^c^	*bla*_NDM-1_	65.38 [3.8875, 1099.53]	0.0037	0.04 [0.02, 0.07]	1.00 [1, 1]
	*bla*_NDM-5_	24.10 [1.33, 437.12]	0.0314	0.02 [0, 0.03]	1.00 [1, 1]
	*bla*_KPC-2_	342.93 [183.69, 640.21]	<0.0001	0.88 [0.84, 0.91]	0.98 [0.97, 0.99]
	*bla*_IMP_	15.24 [0.7847, 295.89]	0.0719	0.01 [0, 0.02]	1.00 [1, 1]
	*bla*_OXA_	0.84 [0.48, 1.46]	0.5302	0.06 [0.03, 0.08]	0.93 [0.92, 0.95]
	*bla*_CTX-M_	4.89 [3.65, 6.55]	<0.0001	0.74 [0.69, 0.79]	0.63 [0.6, 0.67]
	*bla*_CMY_	5.00 [1, 24.88]	0.0494	0.01 [0, 0.03]	1.00 [0.99, 1]
	*bla*_TEM_	9.31 [6.77, 12.8]	<0.0001	0.80 [0.76, 0.84]	0.70 [0.66, 0.73]

a IPM, imipenem.

b beta-lactam resistance genes.

c MEM, meropenem.

### The genome-wide association analysis

**Figure 2:**
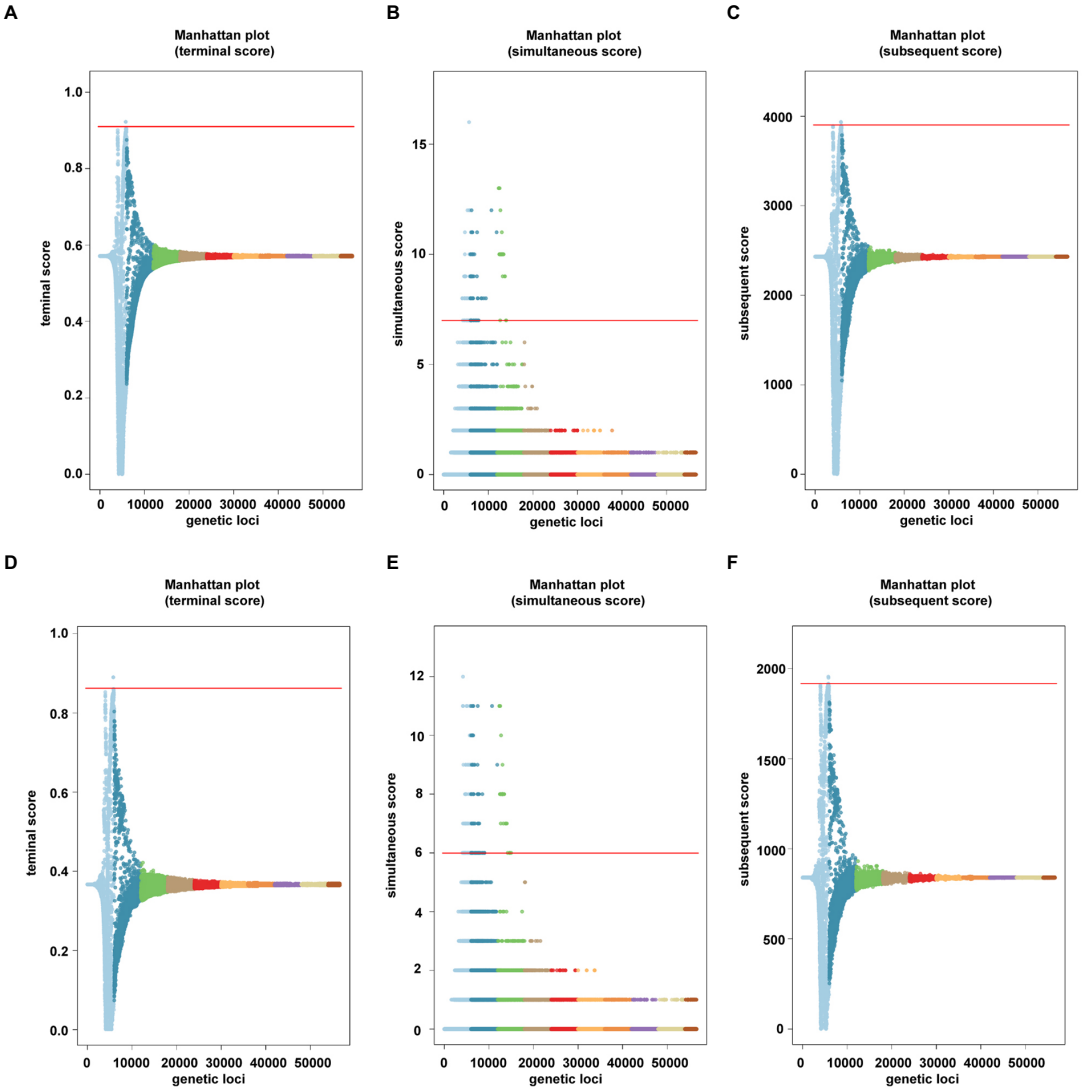
Manhattan plots of the two carbapenems under treeWAS score [**(A–C)** were for IPM, and **(D–F)** were for MEM]. Manhattan plots for **(A,D)** terminal score, **(B,E)** simultaneous score 2, **(C,F)** subsequent score showing association score values for all genes, a significance threshold (red), above which points indicate significant associations.

Genome-wide association was conducted on the 2,033 IPM genomes and 1,017 MEM genomes using a gene or SNP presence or absence matrix with binary phenotype (resistant or susceptible). We identified 28 significant genes associated with the binary result of IPM and 37 significant genes associated with the binary result of MEM. Nine genes were IPM unique and 18 genes were MEM unique. All these related genes and their inference functions (46 genes) are shown in Supplementary Table S3. Nineteen genes were found to be related to both carbapenems. The findings were also shown in the Manhattan figures under three association scores (Figure 2), and the corresponding QQ plot can be found in Supplementary Figure S2.

In 19 genes indentified by both drugs, three genes (dgaR_2, hexR_4, and srlB_2) play a role in glucose catabolism, response regulation, and specificity (Miller et al., 2013), two genes (ompN_1 and tonB_3) are associated with outer membrane pore protein and its interaction with outer membrane receptor proteins, one (cas3) is DNA transferase, one (ubiG_1) is methyltransferase, and one (dppA_4) is ABC transporter peritymal tuberculosis protein. These genes may play an important role in the normal growth and metabolism of bacteria. The unique genes for the two drugs are mostly hydrolases, synthases, and enzymes related to metabolic regulation. For example, folE in IPM is GTP cyclic hydrolase, and guaA_2 is GMP synthase. In MEM, hyuC_2 is the enzyme encoding N-carbamoyl-L—amino acid hydrolase (Supplementary Table S3).

In addition to gene mutations, we also examined the possible influence of SNP variations on carbapenem resistance. We found 13 CR SNPs significantly related to IPM or MEM resistance, nine SNPs of them were found for both carbapenems. Three SNPs were in the non-coding region, 10 SNPs in the coding region with 4 SNPs were synonymous variants and 6 SNPs were missense variants. Four of the six missense variants were identified in both drugs. Among the four SNPs, the SNP in gyrA gene was already reported in the quinolone resistance-determining regions, which was responsible for quinolone-resistant Enterobacteriaceae (Minarini and Darini, 2012). We also found a SNP locate in permease EefB, which was recorded as a multidrug efflux RND transporter in NCBI database. The other three missense variants were located in LysM domain-containing protein, putative DEOR-type transcriptional regulator, and enzyme with alpha/beta-hydrolase domain. Their correlation with resistance warrants further investigation (Table 3).

**Table 3:** SNPs related with resistance to two drugs.

SNP position	Gene locus tag	Function	Variant type	Antibiotic	*p* Value
25,147	KPHS_00200	Putative endonuclease	Synonymous variant	IPM&MEM	<0.01
104,866	Non-coding	/	/	IPM	<0.01
1,900,123	KPHS_18330	Putative amidase	Missense variant	MEM	<0.01
2,959,426	Non-coding	/	/	IPM&MEM	<0.01
3,348,275	KPHS_33660	Hypothetical protein	Synonymous variant	IPM&MEM	<0.01
3,352,661	Non-coding	/	/	IPM&MEM	<0.01
3,356,545	KPHS_33780	Protease 2	Synonymous variant	IPM&MEM	<0.01
3,370,085	KPHS_33920	LysM domain-containing protein	Missense variant	IPM&MEM	<0.01
3,713,110	KPHS_36880	Putative DEOR-type transcriptional regulator	Missense variant	IPM&MEM	<0.01
3,738,471	KPHS_37060 (*gyrA*)	DNA gyrase subunit A	Missense variant	IPM&MEM	<0.01
4,886,999	KPHS_48900	Putative enzyme with alpha/beta-hydrolase domain	Missense variant	IPM&MEM	<0.01
4,923,593	KPHS_49260	Osmolarity sensor protein	Missense variant	MEM	<0.01
5,227,738	KPHS_52090	Multidrug efflux permease EefB	Synonymous variant	IPM	<0.01

The SNPs were identified and annotated using the reference KP genome HS11286 (GCF_000240185.1). The *p* values shown were estimated by three complementary association scores of treeWAS.

### Verification in an independent data set

To validate our findings, the 46 association genes and 13 SNPs were tested in an independent set of KP isolates from the Long et al. study with public sequence and drug resistance phenotypic data. The validation set was downloaded from NCBI SRA, and these KP isolates were cultured from patient specimens in the Houston Methodist Hospital System. Most of the isolates were cultured from urine and respiratory. Different from our data, the ST types were mainly ST258 and ST307 in the validation data set. Forty-five genes of 46 associated genes (except iucD) were detected in the validation set, and 11 genes were significant in the data set. In addition, SNP 104866 in the non-coding region was also well replicated. The results of the fisher’s test were listed in Supplementary Table S3. By querying sting the sequences against the NCBI references, we found that only four genes were located in chromosome, other genes were more likely located in plasmids. In addition, 11 genes were simultaneously validated for both drugs (Table 4). We found that the 11 genes may play a role in different functions related to carbapenem resistance through literature search. The inference functions of the 11 genes were related to biofilm formation or transcriptional regulation of LPS, membrane related antibiotic resistance, UV protection and mutation and other functions resistance related (Table 4). To further confirm our findings, we also performed a fisher test on the combined data of the test and validation data set. The results showed that seven genes in IPM and eight genes in MEM were still significate (Supplementary Table S4).

**Table 4:** Genes and their functions identified in the validation data.

Gene	Position	Inference function	IPM_Odds ratio	IPM_*p* Value	MEM_Odds ratio	MEM_*p* Value
arsD_2	Plasmid/chromosome	Regulatory protein	1.79 [1.36, 2.35]	<0.0001	1.73 [1.31, 2.27]	0.0001
baiA1	Chromosome	Hydroxysteroid dehydrogenases	1.90 [1.43, 2.51]	<0.0001	2.32 [1.75, 3.08]	<0.0001
ecpA_2	Chromosome	Biofilm development	4.46 [3.29, 6.06]	<0.0001	4.18 [3.09, 5.67]	<0.0001
htpX_2	Plasmid/chromosome	Biofilm formation	1.67 [1.28, 2.19]	0.0002	1.94 [1.48, 2.53]	<0.0001
pir	Plasmid	DNA replication	5.94 [3.81, 9.24]	<0.0001	6.91 [4.37, 10.93]	<0.0001
ptlH	Plasmid	Transporter	4.40 [3.20, 6.05]	<0.0001	4.38 [3.18, 6.02]	<0.0001
rfaH_2	Plasmid	Expression of LPS	5.06 [3.64, 7.02]	<0.0001	5.13 [3.69, 7.12]	<0.0001
rrrD_2	Chromosome	Biofilm formation	2.11 [1.34, 3.33]	0.0013	2.11 [1.34, 3.32]	0.0013
umuC_2	Plasmid	UV protection and mutation	2.59 [1.96, 3.44]	<0.0001	2.33 [1.76, 3.09]	<0.0001
ydjE_3	Chromosome	Biofilm formation	2.01 [1.35, 3.00]	0.0006	2.15 [1.45, 3.21]	0.0002
yjoB	Plasmid	ATPase	4.53 [3.27, 6.28]	<0.0001	4.53 [3.27, 6.29]	<0.0001

The genes were identified and annotated by Prokka; The *p* values shown were estimated by fisher test and a gene was considered validated if the *p* value < 0.01 and OR > 1.

## Discussion

Carbapenem resistance in KP is a global challenge often leads to prolonged disease durations and even failure of clinical antimicrobial therapy. Carbapenems are considered as one of the last resort antibiotic to treat multi-drug resistant KP. However, CRKP became more prevalent year by year. In recent years, reports of CRKP have increased worldwide due to the lack of appropriate medical intervention and antibiotic abuse. In order to provide theoretical guidance for the prevention, controlling and intervention of CRKP, we examined 2,033 and 1,017 clinical KP genomes with IPM and MEM binary MIC phenotype using genomic characterization and genome-wide association analysis. High genomics diversity was observed and resistant phenotype explanation for the known carbapenems resistance genes was also discussed. Our GWAS results identified 46 genes that related with resistance and 11 genes were validated in a dependent dataset. These genes have been found to play a role in biofilm formation, transcriptional regulation of lipopolysaccharide and DNA damage repair. No missense mutation SNPs were found significate except one non-coding region SNP probably due to most of the SNPs are strain-specific.

Both imipenem and meropenem belong to carbapenems antibacterial drugs. The main mechanism is to inhibit KP and other *Enterobacteriaceae* strains mediated by the production of a β-lactamases capable of hydrolyzing most β-lactams antibiotics including carbapenems (Reyes et al., 2019). They all have broad-spectrum and strong antibacterial effects and have numeroussimilar properties. However, due to the different chemical structure, there are differences in antibacterial activity and drug resistance between imipenem and meropenem. Our data showed that both drugs had higher resistance rates (21.4 vs. 31.7%, respectively) than reported, and MEM was higher than IPM. In addition, ST11 and KL47 dominated the two datasets. They have been reported to have the majority of CRKP isolates in our previous study. For the known resistant genes encoding carbapenemase, *bla*_KPC_ which is endemic in China (Perez and Van Duin, 2013) is also the most common gene in both IPM and MEM. However, our analysis also showed that all these existing CR genes have high sensitivity, but their low specificity on carbapenem resistance phenotypes means that carbapenem resistance may be mediated by other resistance determinants. Improvements in next-generation sequencing and computational methods such as GWAS can facilitate other resistance determinants identification. Of the 19 genes shared by both drugs in GWAS results, most of them belong to the genes related to bacterial growth and metabolism, and the specific genes may be related to different synthetic, hydrolysis, and metabolic regulation pathways of the two carbapenems. Previous studies have reported in *Escherichia coli* by building machine learning with genome-scale metabolic models and found that bacterial metabolism and the growth of key genes were associated with drug resistance (Pearcy et al., 2021). In our study, we found 12 genes were associated with metabolism and growth, seven genes were also confirmed in an independent dataset. Our results strongly suggested that metabolic adaptations in cell wall, energy, iron and nucleotide metabolism maybe associated with drug resistance.

Biofilm formation is a widely seen phenomenon in bacterial colonization and biofilm can protect the bacteria from attack by the host immune and provide protection from antimicrobial agents (Gupta et al., 2016). Other research also reported that antibacterial resistance had a significant association with biofilm formation in KP isolates (Alcantar-Curiel et al., 2018). LPS also plays a vital role in Gram-negative bacteria in resistance (Nickerson et al., 2017). UV radiation can increase mutation rates and can be used to adjust mutation rates in many applications (Shibai et al., 2017). The horizontal transfer of drug resistance genes is also an essential mechanism of drug resistance acquisition in KP. Our GWAS results highly confirmed these correlations. Firstly, some of these genes play a role in biofilm formation or transcriptional regulation of LPS. The ecpA gene was a part of ecpRABCDE operon, which may have a role in early-stage biofilm development and host recognition (Bhowmik et al., 2014). The rfaH gene product was reported to be required for normal expression of LPS in *E. coli* (Rojas et al., 2001). RrrD was reported in *E. coli* that host may not form wild-type biofilms with its mutants (Toba et al., 2011). Secondly, three genes may have a function in membrane related antibiotic resistance. ArsD was reported as a regulatory protein that confered resistance to arsenic and antimony in *E. coli* (Wu and Rosen, 1993). YdjE has been identified as an inner membrane metabolite transport protein. The htpX gene encoded a putative membrane-bound zinc metalloprotease that may play a role in membrane protein proteolytic quality control (Sakoh et al., 2005). In addition, UmuC gene was involved in UV protection and mutation, and UV can induce bacterial mutations that increase resistance to antibacterial drugs (Tang et al., 2000). Besides the above genes, BaiA was reported to be a 3α-Hydroxysteroid Dehydrogenases that was involved in the network of enzymes that catalyze the hydroxyl group in Clostridium scindens (Shibai et al., 2017). The pir gene was located in plasmid R6K DNA which was an antibiotic plasmid. The PtlH was an essential component of the Ptl system of the type IV transporter that was responsible for secretion of pertussis toxin (PT) across the outer membrane of Bordetella pertussis (Verma and Burns, 2007). YjoB protein exhibited a ATPase activity and was reported that modulate the activity of proteases (Kotschwar et al., 2004). These functions were all crucial in antibiotic resistance.

This study has some strengths. To our knowledge, this is the first and largest genome-wide association study of KP with binary phenotype. The phenotypes and genotypes associated with two important carbapenemases were comprehensively investigated. The study highlights the role of genes in the mechanism of carbapenem resistance and emphasizes the feasibility of discovering a large number of new drug resistance genes by GWAS under the premise of a large number of genomic and phenotypic data.

This study also has limitations. First, we used binary phenotypes for association analysis and did not conduct continuous phenotypes for further mining, so the mechanism of drug resistance caused by quantification could not be found. Second, although we used independent data for verification, the effect of verification may be affected to some extent because the data set was different from our data in terms of region and population structure. And most of these samples were isolated from respiratory secretion which might have affected the type of genes we were able to find. Furthermore, these genes still need further functional and experimental validation.

In conclusion, genomics diversity and population structure of the 2,035 KP clinical isolates were described, the association study with the two carbapenems phenotype have identified potential resistant genes. Our study improved the understanding of the genetic mechanisms of carbapenem resistance in *Klebsiella pneumonia*. It will facilitate drug development, drug abuse prevention and control of carbapenem resistance in clinical bacteria. Even though, the potential benefits of the genome-wide association study will require continued efforts in open sharing of data and tools.

## Data availability statement

The datasets presented in this study can be found in online repositories. The names of the repository/repositories and accession number (s) can be found at: https://db.cngb.org/search/project/CNP0001198/, CNP0001198. Chen et al., 2020, Guo et al., 2020).

## Ethics statement

This study was approved by the ethics committees under tracking numbers of 201806861 and BGI-IRB 18061.

## Author contributions

NP and JL conceptualized the research. WS and NP performed the genomic analysis and wrote the manuscript. JH and YL performed the laboratory work. All authors contributed to the article and approved the submitted version.

## Acknowledments

This work was supported by China National GeneBank (CNGB).

## Conflict of interest

The authors declare that the research was conducted in the absence of any commercial or financial relationships that could be construed as a potential conflict of interest.

## Publisher’s note

All claims expressed in this article are solely those of the authors and do not necessarily represent those of their affiliated organizations, or those of the publisher, the editors and the reviewers. Any product that may be evaluated in this article, or claim that may be made by its manufacturer, is not guaranteed or endorsed by the publisher.

## Footnotes

^1^ https://github.com/tseemann/snippy

## Supplementary material

Supplementary Figure S1 Pan-genome of IPM and MEM dataset.

Supplementary Figure S2 QQ plot of IPM and MEM.
